# Serum pleiotrophin as a diagnostic and prognostic marker for small cell lung cancer

**DOI:** 10.1111/jcmm.14116

**Published:** 2019-01-11

**Authors:** Chunhua Xu, Yuchao Wang, Qi Yuan, Wei Wang, Chuanzhen Chi, Qian Zhang, Xiuwei Zhang

**Affiliations:** ^1^ Endoscopic Center of Nanjing Chest Hospital Nanjing Jiangsu China; ^2^ Clinical Center of Nanjing Respiratory Diseases and Imaging Nanjing Jiangsu China; ^3^ Department of Respiratory Medicine Nanjing Chest Hospital Nanjing Jiangsu China; ^4^ Department of Respiratory Medicine Affiliated Jiangning Hospital of Nanjing Medical University Nanjing Jiangsu China

**Keywords:** diagnosis, pleiotrophin, SCLC, serum

## Abstract

Pleiotrophin (PTN) is involved in tumour progression, angiogenesis and metastasis. The purpose of this study was to investigate the expression level of PTN in the serum of patients with small cell lung cancer (SCLC) and to explore the clinical significance of PTN. Serum samples from 128 patients with SCLC, 120 healthy volunteers (HV) and 60 patients with benign lung disease (BLD) were collected. The levels of serum PTN were determined with ELISA and its correlation with the clinical data was examined. The serum PTN levels in SCLC patients were significantly higher than that in BLD patients (*P* < 0.05) or HV (*P* < 0.05). With a cutoff value of 258.18 ng/mL, the sensitivity and specificity of PTN to SCLC patients and BLD patients, SCLC patients and HV were 79.2% and 91.7%, 86.7% and 95.8% respectively. An area under the curve for all stages of SCLC resulting from PTN, which was significantly better than the other tumour markers tested including progastrin‐releasing peptide and neuron‐specific enolase. High serum PTN levels appear to correlate with poor survival in patients with SCLC. These results suggest that PTN levels in the serum could be a new effective biomarker for the diagnosis and prognosis of SCLC.

**Abbreviations**
BLD: benign lung disease
HV: healthy volunteers
PTN: pleiotrophin
SCLC: small cell lung cancer

## 1 INTRODUCTION

Lung cancer is the leading cause of cancer related deaths worldwide.^1^ Small cell lung cancer (SCLC) accounts for 13% of the total number of lung cancer.^2^ Although patients with SCLC respond significantly to chemotherapy or radiotherapy, most of them die from recurrent diseases.^3^ With early metastasis, more than half of patients with SCLC are extensive diseases.^4^ Therefore, the 5‐year survival rate of SCLC is still very low.^5^ Progastrin‐releasing peptide (ProGRP), YKL‐40 and neuron‐specific enolase (NSE) is commonly used tumor markers for tumor diagnosis.^6^, ^7^, ^8^ However, these widely used serum marker are not sufficiently accurate to be useful as a diagnostic test. Therefore, the search for novel biomarkers remains an important task for SCLC diagnosis.

Pleiotrophin (PTN) is a secretory growth factor with high affinity for heparin.^9^, ^10^ In recent years, PTN activity is believed to stimulate the differentiation of neural stem cells, bone progenitor cells and bone marrow stem cells.^11^, ^12^, ^13^ It is also detected in some tumour cells and tumor specimens, and plays an important role in tumor progression, angiogenesis and metastasis.^14^, ^15^, ^16^ However, there is limited clinical research on the over expression of PTN in lung cancer. Jager et  al detected the expression of PTN in 14 non‐small cell lung cancer (NSCLC) cell lines and 12 SCLC cell lines by RT‐PCR. The results showed that PTN mRNA was expressed in nine SCLC and three NSCLC cells.^17^ In a subsequent small sample study, 87% of SCLC patients and 63% of NSCLC patients had elevated serum levels of PTN, suggesting the potential role of serum PTN levels in the diagnosis of lung cancer.^18^ Recent studies suggest that serum PTN may be a useful marker for the diagnosis and prognosis of lung cancer.^19^ Pleiotrophin is overexpressed in SCLC tissues and the levels of PTN expression are associated with the diagnosis and prognosis.^20^

However, the potential role of serum PTN levels in early diagnosis and prognosis of lung cancer, especially SCLC, a type of lung cancer, still requires further investigation.

In this study, we first examined serum PTN levels in patients with SCLC with an ELISA kit. Additionally, we investigate the correlation of PTN levels and adverse clinic features of SCLC such as tumor stage. Our results show that an elevated level of serum PTN serves as both a poor clinical state and a valuable diagnostic and prognostic biomarker for SCLC.

## 2 METHODS

### 2.1 Patients

We consecutively enrolled 128 patients with SCLC, 60 patients with benign lung disease (BLD), and 120 healthy volunteers (HV). Serum samples from all subjects were collected at Nanjing chest hospital. Patients with SCLC were included if they met the following criteria: confirmation of SCLC via a review of pathologic slides by two independent observers to classify the histologic subtype; no pro‐surgical or pro‐diagnostic history of antineoplastic therapy, radiotherapy, or chemotherapy. Before treatment, the patients were divided into limited stage or extensive SCLC. Limited disease is defined as a single radiation field that is limited to the ipsilateral chest, and widespread disease is defined as a disease other than the ipsilateral half thorax, including malignant pleural or pericardial effusion or blood transfer. Contralateral mediastinum and ipsilateral supraclavicular lymphatic diseases are divided into limited stages. Sublateral supravalinal lymphnode disease and supraclavicular lymphnode disease are often classified as extensive diseases.^21^ All the patients in this study received platinum‐based chemotherapy combined with errisecan or etoposide. The characteristics of the SCLC patients are presented in Table 1. Follow‐up information was obtained by phone investigations. The median follow‐up of surviving patients at the time of analysis was 12 months (range, 6‐30 months). The date of the last follow‐up was March 21, 2017. Overall survival (OS) was defined as the time interval between the date of diagnosis and the date of death or the last follow‐up.

**Table 1:** The characteristics of patients with SCLC, patients with BLD and HV

Variables	SCLC	BLD	HV	*P*‐value
Subject, NO	128	60	120	
Age, y	58.7 ± 12.8	57.6 ± 11.6	57.8 ± 12.5	>0.05
Male/female	60/68	35/25	68/52	>0.05
SCLC				
Limited	58			
Extended	70			
BLD				
Tuberculosis		30		
Bronchiectasis		10		
CAP		20		

BLD, benign lung disease; CAP, community acquired pneumonia; HV, healthy volunteers; SCLC, serum of patients with small cell lung cancer.

Patients with BLD were identified via CT screening, etiology, response to antibiotics and subsequently monitored for 6 months using CT, with no evidence of cancer. None had a history of previous cancer or chemotherapy. Healthy volunteers were subjects who had not received a diagnosis of malignant or benign disease after routine examinations, including CT, ultrasonographic examination, and routine laboratory tests. The baseline characteristics of patients with BLD and HV are presented in Table 1.

All participants provided written informed consent, and the Nanjing Chest Hospital Ethics Committee approved the research project.

### 2.2 Serum collection

Blood samples from 128 patients with SCLC were obtained from the patients after diagnosis but prior to any treatment. After two cycle of chemotherapy, serum samples were obtained again. The whole blood samples were promptly centrifuged at 1500 *g* for 15 minutes and the supernatant stored at −80°C until use.

### 2.3 Determination of PTN, ProGRP and NSE levels

The serum concentration of PTN was determined by using a commercial, two‐site, sandwich‐type ELISA (eBioscience, Santiago, USA) using streptavidin‐coated microplate wells, a biotinylated‐Fab monoclonal antibody, and an alkaline phosphatase‐labeled polyclonal detection antibody. Serum NSE levels were measured by commercial ELSA‐NSE kits (CIS Bio International, Gif‐Sur Yvette, France); serum ProGRP was determined by ELISA Kit (ALSI, IBL‐ Hamburg, Germany). The intra‐assay coefficient of variation (CV) was <5%, and the interassay CV was <6%. The normal upper limit of tumor markers is ProGRP 46 pg/mL and NSE 13 ng/mL. All tests were done in two copies and diluted properly, and technicians ignored the clinical data.

### 2.4 Statistical analysis

Statistical software (spss for Windows, version 18) was used for data analysis. All values are given as mean ± SD was used. The Mann‐Whitney *U* test was used to compare between serum sample groups, and the Kruskal‐Wallis test was used to compare several groups. Chi square test was used to test the correlation between serum PTN and clinical parameters. Receiver operating characteristics (ROC) analysis was plotted to determine the sensitivity and specificity of serum PTN levels to differentiate SCLC from BLD as well as SCLC and HV. The diagnostic power of serum PTN was assessed by sensitivity, specificity, and area under ROC curve (AUC). The cutoff value was determined by the score closest the value under both peak of sensitivity and specificity. Survival curves were plotted by the Kaplan‐Meier method and compared using the log‐rank test. Binary logistic regression was used to assess whether the diagnostic efficiency of PTN in combination with ProGRP and NSE was superior to that of the three individual biomarkers alone. The value of *P* < 0.05 has statistical significance.

## 3 RESULTS

### 3.1 Serum PTN, ProGRP and NSE levels in SCLC patients

**Figure 1:**
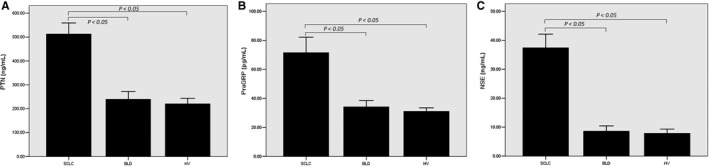
Levels of pleiotrophin (PTN), progastrin‐releasing peptide (ProGRP) and neuron‐specific enolase (NSE) in three groups. Among 128 small cell lung cancer (SCLC) patients, the serum levels of PTN (A), ProGRP (B) and NSE (C) were significantly higher than those of benign lung disease (BLD) group and healthy volunteers (HV) group (*P* < 0.05).

As shown in Figure 1A, serum PTN levels in SCLC group were significantly higher than those in BLD group or HV group (*P < *0.05). Serum PTN levels in HV group were similar to those in BLD group (220.26 ± 41.59 ng/mL vs 239.39 ± 46.44 ng/mL, *P* > 0.05). The levels of serum ProGRP and NSE in patients with HV, BLD and SCLC were also shown in Figure 1B,C. Compared with HV and BLD, serum ProGRP and NSE levels in SCLC patients also increased significantly (*P < *0.05).

### 3.2 Diagnostic value of serum PTN

**Figure 2:**
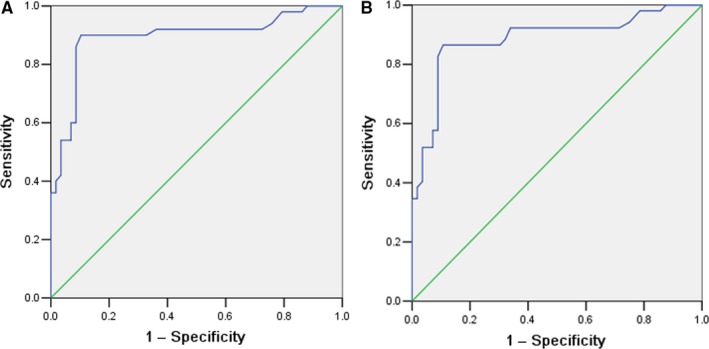
Receiver operating characteristics analysis of pleiotrophin for differentiation of patients with small cell lung cancer (SCLC) from healthy volunteers (HV) (A) and from patients with benign lung disease (BLD) (B). The analysis resulted in an area under the curve of 0.894 (patients with SCLC vs. HV) and 0.885 (patients with SCLC vs patients with BLD), respectively

The sensitivity of the index in distinguishing SCLC patients, HV and BLD patients was calculated. As shown in Figure 2A,B, the area under curve of serum PTN was 0.894 and 0.885 respectively. With a cutoff value was 258.18 ng/mL, the sensitivity and specificity of PTN to differentiate SCLC from BLD, SCLC and HV was 79.2%, 91.7%, 86.7% and 95.8%, respectively. It is suggested that serum PTN is a valuable biomarker for the diagnosis of SCLC.

**Figure 3:**
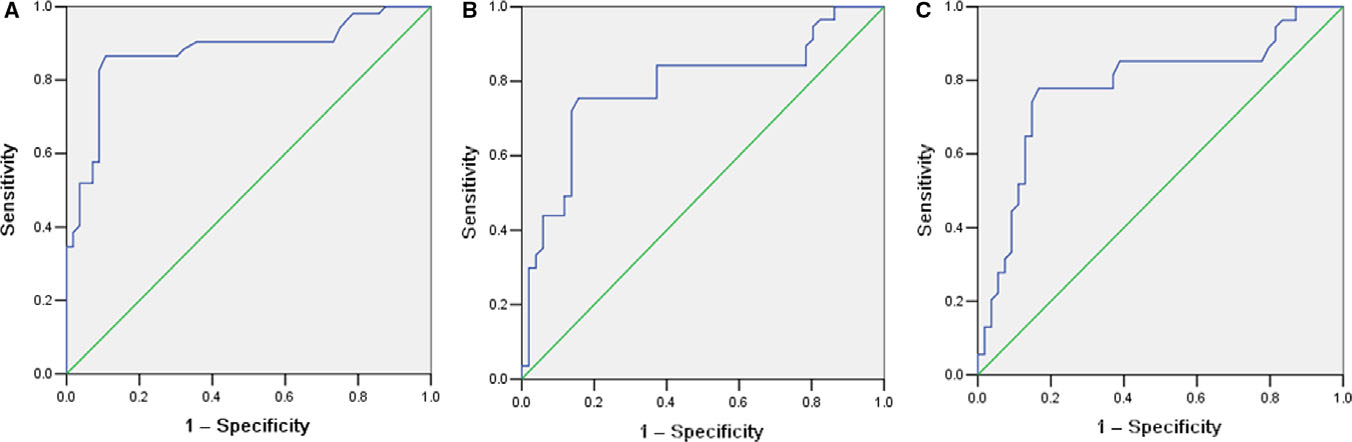
Comparison of pleiotrophin (PTN) and other tumor marker on diagnosis for small cell lung cancer. Receiver operating characteristics analysis resulted in an area under the curve of 0.887, 0.784 and 0.763 for PTN (A), for progastrin‐releasing peptide (B) and for neuron‐specific enolase (C), respectively

ROC curves were plotted to determine the diagnostic efficiency of serum PTN levels for SCLC. The efficiency of the ProGRP and NSE, in distinguishing SCLC from sex/age‐matching the controls was also included. The measurements of the different individual markers and their predictive value in the diagnosis of SCLC are summarized in Table 2. An AUC for SCLC resulting from PTN (0.887), which was significantly better than the other tumor markers tested including ProGRP (0.784)and NSE (0.763) (Figure 3A‐C and Table 2). These results suggest serum PTN is a high performance biomarker for SCLC.

**Table 2:** The diagnostic efficiency of models in differentiating SCLC patients and the controls

SCLC vs control	AUC (95% Cl)	SN (%)	SP (%)	PPV (%)	NPV (%)
PTN^a^	0.887 (0.806‐0.948)	70.3	94.4	90.9	81.8
ProGRP	0.784 (0.694‐0.874)	60.2	82.2	70.6	74.4
NSE	0.763 (0.669‐0.857)	49.2	86.7	72.4	70.6
PTN + ProGRP + NSE	0.914 (0.857‐0.971)	80.5	98.9	98.1	87.7

AUC, areas under the curves; NPV, nagative predictive value; NSE, neuron‐specific enolase; PPV, positive predictive value; ProGRP, progastrin‐releasing peptide; PTN, pleiotrophin; SN, sensitivity; SP, specificity; SCLC, serum of patients with small cell lung cancer.

a The diagnostic cut‐off value was 258.18 ng/mL.

We used binary logistic regression to investigate whether combining the markers could improve diagnostic accuracy. The combination of PTN, ProGRP and NSE improved the classification capacity and yielded a better optimal diagnostic efficacy for SCLC patients (Table 2) than did DDH2 alone (*P* < 0.01).

### 3.3 Relationship between PTN levels and clinicopathological characteristics

A cutoff point of 258.18 ng/mL, the patients were divided into high PTN and low PTN. The relationship between serum PTN level and clinicopathological characteristics in patients with lung cancer was nanlyzed. As shown in Table 3, PTN levels are associated with stage of disease (*P < *0.05). However, there was no significant correlation between PTN level and age, sex, performance status and smoking status (*P* > 0.05).

**Table 3:** Relationship between PTN levels and clinicopathological characteristics

Characteristics	Total	Serum PTN		
		High^a^	Low^b^	*P*‐value
Age, y				0.472
≥60	70	38	32	
<60	58	36	22	
Gender				0.373
Male	60	32	28	
Female	68	42	26	
Smoking status				0.211
Non‐smoker	72	38	34	
Smoker	56	36	20	
Performance status				0.462
0‐1	80	44	36	
2‐3	48	30	18	
Disease stage				0.001*
Limited	58	24	34	
Extended	70	50	20	

PTN, pleiotrophin.

a High group represents the levels of serum PTN ≥ 258.18 ng/mL.

b Low group represents the levels of serum PTN < 258.18 ng/mL.

* Statistically significant difference (*P* < 0.05)

### 3.4 Serum PTN and chemotherapy response

To evaluate the effect of serum PTN level on the efficacy of chemotherapy, data from SCLC patients were collected. There was significant difference between serum PTN levels before and after chemotherapy for SCLC patients, which is (512.56 ± 99.34) ng/mL and (380.91 ± 97.87) ng/mL respectively (*P < *0.05). The level of serum PTN before treatment has an effect on response to chemotherapy. Before treatment, the level of serum PTN (442.16 ± 98.64 ng/mL) in complete or partial response was significantly lower than that of patients with stable or progressive disease (635.58 ± 102.76 ng/mL, *P < *0.05).

### 3.5 Association of serum PTN levels with OS of SCLC patients

**Figure 4:**
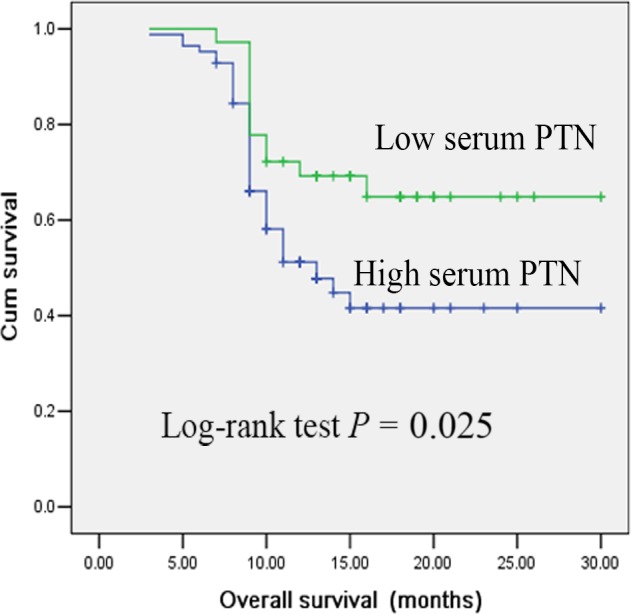
Kaplan‐Meier survival analysis of patients with small cell lung cancer (SCLC) based on serum pleiotrophin (PTN) levels. The overall survival rate of SCLC patients with high serum PTN level was significantly lower than patients with low serum PTN level (*P* = 0.025; log‐rank test)

Next, we examined the association between serum PTN levels with OS of patients with SCLC by performing the Kaplan‐Meier survival analysis. We found that patients with higher levels of serum PTN level had significantly worse OS rates (*P* = 0.025, Figure 4).The follow‐up time of these 120 patients was from 6 to 30 months, with a medium time of 12 months.

## 4 DISCUSSION

Pleiotrophin is overexpression in many tumours and affects many aspects of tumour biology. Previous studies have shown that PTN can be considered as a diagnostic and prognostic indicator for NSCLC. However, the diagnostic value of serum PTN level for SCLC has not been extensively studied. The purpose of this study was to investigate the diagnostic value of serum PTN level in patients with SCLC.

Pleiotrophin is a secreted cytokine related to diverse biological properties, including neuritis outgrowth, angiogenesis, and tumour growth. It is strongly expressed in different human tumour cells, and expression of the PTN gene in tumour cells in vivo accelerates growth and stimulates tumour angiogenesis. Based on previous results, overexpression PTN could be detected in a wide variety of human cancers, and is believed to be proto‐oncogenic. In vitro and in vivo experiments confirmed that elevated expression of PTN could increase the malignant grade of tumor cells, whereas knockdown of PTN can effectively inhibit tumour cell growth, migration and invasion.^22^

We have described for the first time the expression of serum PTN in patients with SCLC and BLD. Compared with serum PTN levels in HV or patients with BLD, serum PTN levels in patients with SCLC increased significantly, making it a potential auxiliary tool for diagnosing SCLC. Importantly, we found that PTN was significantly correlated with disease stages, suggesting that PTN may be a valuable marker for predicting tumour progression in SCLC patients. Du et al reported the relationship between serum PTN and stage.^19^ Their results can be considered similar to ours.

Previous studies had reportedthe PTN is a tumour marker in NSCLC, with a sensitivity and specificity of 78.4% and 66.7% at cutoff value of 300.1 ng/mL.^19^ In the present study, the diagnostic value was 258.18 ng/mL and had a sensitivity and specificity of PTN to differentiate SCLC from BLD, SCLC and HV was 79.2%, 91.7%, 86.7% and 95.8%, respectively. As shown in Figure 2, serum PTN is a valuable marker for differentiating SCLC patients from BLD patients and HV. To further assess the potential of serum PTN as a diagnostic marker of SCLC, we will compare it with ProGRP and NSE. The AUC values of ProGRP, NSE and PTN in SCLC vs the controls were 0.784, 0.763 and 0.887, respectively. It is suggested that serum PTN is a good biomarker for the diagnosis of SCLC. We also tested ProGRP and NSE levels and compared their diagnostic efficacy to that of PTN. The results indicated that combined detection of these four markers had a better diagnostic value than that of single marker for the discrimination of SCLC from the controls. This may offer a new method in differentiating SCLC patients and the controls.

In this study, thirty high PTN patients did not respond to chemotherapy. Therefore, high PTN is most often associated with chemotherapy resistant patients. These patients should be good candidates for evaluating clinical trials of alternative therapies. Moreover, PTN levels were significantly decreased after chemotherapy, especially in the complete or partial response patients. The level of serum PTN was found to be an indicator of poor survival and could serve as a prognostic biomarker for patients with SCLC. Importantly, it might be served as an effective and accurate biomarker for evaluating the chemotherapy outcome of patients with SCLC.

The findings of this study support our hypothesis that serum PTN is a promising serum biomarker which may help to improve SCLC diagnosis and prognostic assessment. However, further studies in large, well‐characterized patient samples are needed to confirm and expand our findings. As more biomarkers are discovered and validated, efforts will be focused on identifying an appropriate set of markers, including serum PTN, which can increase the sensitivity and accuracy of detecting early SCLC. The increase in sensitivity and specificity conferred by such a panel could potentially have a significant impact on the survival of patients with lung cancer.

## 5 CONCLUSIONS

In conclusion, we have confirmed the increase of serum PTN level in patients with SCLC in this study. High PTN levels appeared to correlate with poor survival in patients with SCLC. These results suggested that serum PTN could be a new effective biomarker for the diagnosis and prognosis of SCLC. Further studies are needed to clarify the precise role of PTN in tumor growth, and more large‐scale prospective studies are needed to confirm this finding.

## CONFLICT OF INTEREST

The authors declare no any conflicts of interest in this work.
